# Identifying the genetic basis of viral spillover using Lassa virus as a test case

**DOI:** 10.1098/rsos.221503

**Published:** 2023-03-22

**Authors:** Alexander O. B. Whitlock, Brian H. Bird, Bruno Ghersi, Andrew J. Davison, Joseph Hughes, Jenna Nichols, Matej Vučak, Emmanuel Amara, James Bangura, Edwin G. Lavalie, Marilyn C. Kanu, Osman T. Kanu, Anna Sjodin, Christopher H. Remien, Scott L. Nuismer

**Affiliations:** ^1^ Department of Biological Sciences, University of Idaho, Moscow, ID, USA; ^2^ Department of Mathematics and Statistical Science, University of Idaho, Moscow, ID, USA; ^3^ One Health Institute, School of Veterinary Medicine, University of California, Davis, Davis, CA, USA; ^4^ MRC-University of Glasgow Centre for Virus Research, Glasgow, UK; ^5^ University of Makeni and University of California, Davis One Health Program, Makeni, Sierra Leone

**Keywords:** genome-wide association studies, Lassa virus, zoonosis, single-nucleotide polymorphisms, spillover, host shift

## Abstract

The rate at which zoonotic viruses spill over into the human population varies significantly over space and time. Remarkably, we do not yet know how much of this variation is attributable to genetic variation within viral populations. This gap in understanding arises because we lack methods of genetic analysis that can be easily applied to zoonotic viruses, where the number of available viral sequences is often limited, and opportunistic sampling introduces significant population stratification. Here, we explore the feasibility of using patterns of shared ancestry to correct for population stratification, enabling genome-wide association methods to identify genetic substitutions associated with spillover into the human population. Using a combination of phylogenetically structured simulations and Lassa virus sequences collected from humans and rodents in Sierra Leone, we demonstrate that existing methods do not fully correct for stratification, leading to elevated error rates. We also demonstrate, however, that the Type I error rate can be substantially reduced by confining the analysis to a less-stratified region of the phylogeny, even in an already-small dataset. Using this method, we detect two candidate single-nucleotide polymorphisms associated with spillover in the Lassa virus polymerase gene and provide generalized recommendations for the collection and analysis of zoonotic viruses.

## 1. Introduction

Zoonotic disease imposes a massive toll on human health, sickening as many as 2.5 billion people annually and causing 2.7 million deaths each year [1]. In the wake of COVID-19, the threat of zoonotic viruses has become even more palpable, and recent studies suggest this threat will only increase as climate change and human range expansion disrupt stable ecosystems and bring humans and domesticated animals into increased contact with wildlife [2]. As humans and wildlife come into contact more frequently, opportunities for zoonotic viruses to spill over into the human population grow, and the potential for novel human viruses to emerge increases [3–5].

Although the traditional approach to managing emerging infectious disease focuses on curtailing outbreaks within the human population after spillover has occurred, this approach does not always succeed. Consequently, interest continues to grow in more proactive approaches that focus on reducing the risk of spillover before it can seed outbreaks, epidemics or pandemics within the human population [6,7]. For instance, it may be possible to reduce the threat of disease by reducing contact between humans and reservoirs [8] or reducing virus prevalence through wildlife vaccination [9]. Because these efforts can be challenging and costly to implement, they can be used most effectively if areas with a high risk of virus spillover can be identified *a priori*. This realization has motivated efforts to identify regions of high risk based on environmental and population features [1,10]. Little progress has been made, however, in identifying viral genotypes that promote spillover into the human population. If such high-risk viral genotypes could be identified, it would open the door to using genomic surveillance to refine our understanding of spillover risk over space and time.

In principle, the detection of virus genotypes capable of spillover can be accomplished by comparing human-derived viral samples with samples collected from the animal reservoir. This approach is directly analogous to the use of genome-wide association studies (GWAS) to identify sites in the genome associated with a particular trait. Typically, GWAS uses large sample sizes to compare paired populations with large genomes and relatively low genetic divergence between samples. For most zoonotic viruses, the available genetic data has none of these advantages. Sample size may be limited, especially for samples collected from wildlife, and human-derived samples may have been collected from different regions than samples derived from the animal reservoir [11,12]. The substitution rate in viruses also far exceeds that in vertebrates [13].

When samples are collected unevenly from a genetically diverse population, the groups under comparison may differ both in their genetic background as well as in the trait of interest. This phenomenon is known as population stratification. Consider, for example, a dataset in which the human-derived viral samples were collected from a different region than the zoonotic samples. A variant that is only found in human samples might be truly associated with human infection, or it might simply be the product of spatial patterns of genetic variation. Population stratification increases the likelihood that GWAS will produce spurious associations due to shared ancestry [14].

Ideally, population stratification would be resolved by collecting additional data to produce a dataset in which human- and animal-derived viral samples are paired [15]. In practice, the cost and difficulty of sample collection for many zoonotic pathogens make this an unrealistic solution [16]. Here, we pursue a more practical solution that corrects for population stratification using information on ancestry encoded within the genome of pathogen samples. Specifically, we use EIGENSTRAT, a form of principal component analysis (PCA) that detects and corrects for patterns that are produced by shared ancestry [17,18]. EIGENSTRAT was developed within the context of human datasets that generally have larger sample sizes and less significant stratification than observed for zoonotic pathogens. It has never been applied to spillover data to the best of our knowledge, and it is unknown how well it will perform under such extreme conditions.

Here, we evaluated the feasibility of using the EIGENSTRAT approach to identify sites in the viral genome associated with spillover into the human population. We began by analysing simulated datasets with sample sizes and levels of stratification informed by data available for zoonotic pathogens. We used these simulated datasets to predict error rates as a function of population stratification and sample size. Next, we validated these predictions and quantified error rates using a hybrid approach that paired simulations with data from Lassa virus (LASV), a rodent-borne virus endemic to West Africa that is considered to be at high risk of emergence [19]. Finally, we applied our approach to the detection of human-associated single-nucleotide polymorphisms (SNPs) in Lassa virus.

## 2. Methods

### 2.1. Association analyses

Our approach is based on the EIGENSTRAT method developed by Price *et al*. [17]. EIGENSTRAT uses PCA to model genome-wide patterns of genetic variation to detect ancestral differences between the groups under comparison. These patterns are applied as a correction to reduce the impact of population stratification. By correcting for stratification, the method reduces the frequency of false positives caused by spurious associations between SNPs and the trait of interest [17]. Because prior expectations for SNPs involved in human infection will not generally be available for zoonotic pathogens, we used all SNPs to quantify population structure when analysing both real and simulated data.

We conducted EIGENSTRAT PCA using EIGENSOFT v. 6.1.4 to detect SNPs associated with spillover into the human population in both simulated and real datasets following the protocol developed previously [17]. In brief, we first calculated a nucleotide consensus sequence for the genomes being analysed. At each nucleotide position, the major allele frequency was defined as the frequency of the consensus nucleotide, and the minor allele frequency was defined as the summed frequency of all other nucleotides. If there was more than one allele at that site, the site was categorized as an SNP. Because EIGENSTRAT is designed for diploid data, and many viral zoonotic pathogens, including LASV, are haploid, we treated all loci as homozygous for either the major allele or a minor allele. Otherwise, we used EIGENSTRAT’s default parameters with two principal components (PCs). Increasing the number of PCs up to 10 did not qualitatively affect the results of our analyses of simulated data and had no impact on our analyses of LASV sequences. No outlier individuals were detected in the LASV data. We used *p* < 0.0001 as the significance threshold.

### 2.2. Quantifying population stratification

**Figure 1.**
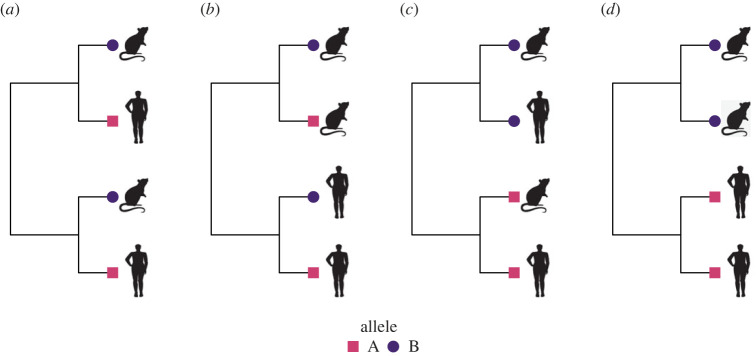
(*a*) Viral samples were collected evenly over the phylogeny, and genetic divergence was low. The frequency of alleles would be expected to be similar in human- and rodent-derived samples. If the allele frequency at a given locus deviated from that expectation, it would indicate a species association. (*b*) Viral hosts were sampled unevenly, which is expected to introduce population stratification, but the severity would be tempered by the low genetic divergence. (*c*) Genetic divergence was high, but the samples were collected evenly across the phylogeny. The distribution of alleles would be similar in human and rodent samples. (*d*) Samples were collected unevenly in a genetically divergent population, producing severe population stratification.

Because one of our primary goals was to investigate how population stratification influences the performance of the EIGENSTRAT approach, we began by quantifying the degree of stratification observed in our LASV dataset. We used the results to ensure that our simulations explored regions of parameter space that produce levels of stratification consistent with zoonotic pathogens. Our general approach to quantifying and simulating population stratification was phylogenetic. Specifically, we quantified population stratification using Pagel’s *λ* [20], a measure of phylogenetic signal. We applied Pagel’s *λ* in two ways, each of which measures a different source of stratification. First, we quantified genetic stratification by calculating Pagel’s *λ* for individual SNPs. This source of stratification, denoted by *λ*_*A*_, measured how evenly an allelic state is distributed over the tips of the viral phylogeny. Second, we quantified host sampling stratification by calculating Pagel’s *λ* for viral host species. This source of stratification, denoted by *λ*_*S*_, measured how evenly host state (i.e. humans versus reservoir) is distributed over the tips of the viral phylogeny. A *λ* value of 0 means adjacent tips of the viral phylogeny are no more likely to carry the same SNP allele or be drawn from the same host species than are distantly related tips (e.g. figure 1*a*). As the value of *λ* approaches 1, adjacent tips of the viral phylogeny become increasingly likely to be drawn from the same host species (e.g. figure 1*b*) or carry the same SNP allele (e.g. figure 1*c*). Overall, stratification is greatest, and the challenge for genetic association methods maximized, when both measures of *λ* are high (e.g. figure 1*d*).

### 2.3. Simulating viral sequences and spillover events

We first explored how population stratification and sample size influenced the performance of the EIGENSTRAT method by simulating viral sequences sampled from humans and animal reservoirs. Our approach began by simulating a viral phylogeny using the function sim.bdtree in the R package geiger [21], which simulates trees under a birth–death process. Trees were produced with either 100 or 1000 tips. For each tip, equivalent to a sample, we simulated a genome with 1000 loci. The allelic value of each locus was simulated independently using the geiger function sim.char, which uses a continuous time Markov chain to evolve a binary character state (equivalent to a biallelic locus) over a phylogeny. We manipulated the strength of phylogenetic signal by using a range of transition rates—0.1, 1, 10 and 100—during evolution. When the transition rate was high, both forward and backward mutations were common. This decreased phylogenetic signal by decreasing the likelihood that descendants from a common ancestor would share the same allele. When the transition rate was low, phylogenetic signal was high because mutations were rare, and entire clades would typically share the same allele. The same transition rate was used for all loci. The host species of each tip in the viral phylogeny was simulated in the same way as allelic state but using an independent transition rate. The frequency of human samples was constrained to be between 35% and 65%.

A genetic basis for spillover risk was simulated by assuming that a single locus determined the likelihood of human infection. We simulated the influence of this locus on the likelihood of human infection by changing the state of each ancestral allele carried by a human sample to the derived allele with a probability equal to *ϕ* as the final step in our simulation protocol. This procedure modelled a scenario where the allele facilitating spillover is neutral within the reservoir population but increases the probability of human infection.

Simulated datasets were analysed by applying EIGENSTRAT to detect associations between SNPs and host species. The Type II error rate (false negative), was measured as the frequency of simulations in which the human-associated locus was not detected. The Type I error rate (false positive) was measured as the frequency with which any of the first 200 background sites in the genome—not involved in spillover risk—was erroneously detected as significant.

### 2.4. Phylogenetic analysis of Lassa virus

Before using EIGENSTRAT to test for genetic associations in the LASV dataset, we generated a phylogeny for the LASV samples and used it to quantify the extent of stratification. LASV is a negative polarity, single-stranded arenavirus with an RNA genome consisting of two segments, each containing two genes. The L segment encodes a small zinc-binding protein (Z) and an RNA-dependent RNA polymerase (L). The S segment encodes two structural proteins, nucleoprotein (NP) and glycoprotein (GPC). LASV accessions were retrieved from GenBank using the search term ‘Lassa Mammarenavirus[ORGN]’. Accessions were removed if they lacked annotated coding regions, had more than two annotated coding regions, or had a synthetic origin. The remaining accessions were re-annotated to standardize segment and gene labels. The amino acid sequences were extracted from each LASV record, and multiple sequence alignments (MSAs) were obtained using Mafft v. 7.475 [22] under default settings. These amino acid MSAs informed codon-guided alignments of the nucleotide sequences for each gene using PAL2NAL v. 13 [23]. To confine our analysis to a single viral lineage, lineage IV, the resulting nucleotide MSAs were filtered to remove any sequences not from Sierra Leone, with the exception of a small number from Guinea and Liberia to be used as an outgroup. This was done because the nucleotide diversity of LASV varies by approximately 30% across lineages [24], and there are few sequences from *Mastomys natalensis* from outside of Sierra Leone, removing the basis for comparison between human and rodent samples. Each nucleotide MSA was used to infer a phylogenetic tree with bootstrap support values using RAxML-NG v. 1.1. [25]. Trees were pruned to remove sequences from non-human and non-*M. natalensis* hosts using the ete3 v. 3.1.2 Python library [26] and visualized using FigTree v. 1.4.4.

The following samples, representing the L and S segments from 15 rodents, were collected from *M. natalensis* as part of this study: OM735989, OM735988, OM735987, OM735986, OM735985, OM735984, OM735983, OM735982, OM735981, OM735980, OM735979, OM735978, OM735977, OM735976, OM735973, OM735972, OM735971, OM735970, OM735969, OM735968, OM791219, OM791220, OM791221, OM791222, OM791227, OM791228, OM791229, OM791230, OM791231, OM791232. All work with wildlife animals was reviewed and approved by the University of California, Davis Institutional Animal Care and Use Committee (IACUC; protocol #22696 Field surveillance for rodent-borne pathogens in West Africa (Sierra Leone); and under the permission of, and in collaboration with, the Sierra Leone Ministry of Health and Sanitation, and the Ministry of Agriculture and Forestry under permit #CONF/LSD/02/17). All other samples were sourced from GenBank [24,27–31]. The full list of GenBank accession numbers with associated metadata is provided in electronic supplementary material, tables S1 and S2. The final dataset contained 67 human samples and 25 rodent samples for the L segment, and 83 humans samples and 30 rodent samples for the S segment.

The phylogeny could be divided into three clades, A, B and C. Viral hosts were distributed unevenly over the phylogeny, and *λ*_*S*_ exceeded 0.99. This was largely driven by Clade C, which contained nearly half of the rodent samples and only one human. To the extent of our knowledge, the samples in Clade C were collected in a different region than those in Clades A and B. Without Clade C, *λ*_*S*_ dropped to 0.52.

### 2.5. Lassa virus-informed simulations

To test the utility of EIGENSTRAT when applied to a zoonotic pathogen with significant impacts on human health, we appended a simulated locus to the polymerase gene sequence of each real LASV sample. Thus, we maintained the phylogenetic structure of the real LASV samples as well as the distribution of hosts from which the LASV sequences were collected. As in the previous simulations, we used sim.char to simulate the allelic state of the appended site as it evolved over the real LASV phylogeny, using transition rates of 1, 10 and 100. In simulations measuring Type II error, we added a human association, *ϕ*, to the artificial locus in the same manner as in the prior section; otherwise, there was no association. We used three values of *ϕ*: 0.25, 0.5 and 0.75. This analysis allowed us to directly evaluate the model’s predictions within the LASV parameter space, namely a small sample size and high *λ*_*S*_.

Next, we performed EIGENSTRAT on the LASV sequences with the artificial simulated locus appended. We measured the Type II error rate as the frequency of simulated datasets in which the appended site was not detected despite its being associated with human infection, and we measured the Type I error rate as the frequency of simulated datasets in which the site was detected as significant despite there being no association. We used 6000 replicates per *ϕ* to measure Type II error and 60 000 replicates to measure Type I error.

To investigate how reducing stratification in the LASV data influenced performance, we analysed both the full dataset and a subset with Clade C removed. We removed Clade C because our initial analyses demonstrated that it was a major source of stratification, particularly with respect to *λ*_*S*_.

### 2.6. Statistical analysis

To assess the contributions of factors driving the Type I and Type II error rates in the simulated datasets, we conducted generalized linear models (GLMs) using a binomial link function. The response variable, significance, was measured on a per-SNP basis and was defined as whether or not the individual SNP was identified as significant by EIGENSTRAT. It was coded as a binary value 0 (not significant) or 1 (significant). When there was no association between the locus and the host species, significance indicated a Type I error. When there was a true association (i.e. *ϕ* > 0), lack of significance indicated a Type II error. We used stepwise selection to produce candidate models, which we evaluated using the R package DHARMa [32], which calculates residuals by simulating new response data. We adjusted the models and fitted restricted cubic splines to address deviations, followed by re-evaluation. The final model was selected by the Akaike information criterion (AIC).

For the simulated datasets, the selected predictors were *λ*_*S*_, *λ*_*A*_ and the frequency of the derived allele in the population. The interaction term was *λ*_*A*_ × allele frequency. Due to high overdispersion in the data measuring Type I error, we used a quasi-binomial link function and quasi-AIC.

For the LASV-informed simulations, the selected predictors were *λ*_*A*_, the frequency of the derived allele in the population, and the data source (categorical with two levels: full data or data subset with Clade C excluded). The interaction term was *λ*_*A*_ × allele frequency. Summaries of each GLM are provided in electronic supplementary material, tables S3–S19.

## 3. Results

### 3.1. Population stratification increased the Type I error rate

EIGENSTRAT analyses of simulated LASV sequence data and spillover events allowed us to study how sample size and population stratification influenced Type I and Type II error rates. As described in the Methods, we quantified two forms of population stratification using Pagel’s *λ* and considered sample sizes of 100 and 1000.

**Figure 2.**
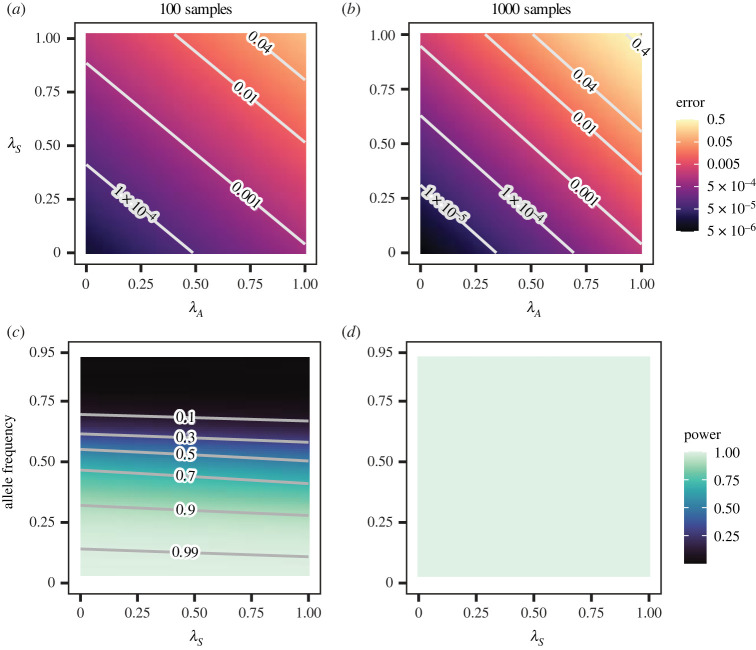
(*a*,*b*) Type I error increased exponentially with population stratification in both sample sizes. (*a*) 100 samples. (*b*) 1000 samples. Each figure represents 3 200 000 SNPs pooled from 16 000 replicate simulations. (*c*,*d*) A large sample size eliminated Type II error. (*c*) 100 samples. (*d*) 1000 samples. Each figure represents the associated SNP from 16 000 replicates. Allele frequency was set to the mean value of 0.5 in (*a*) and (*b*). *λ*_*S*_ was set to the mean value of 0.04 in (*c*).

We found that the Type I error rate increased exponentially with both forms of population stratification, *λ*_*S*_ and *λ*_*A*_, in both sample sizes (figure 2*a*,*b*, electronic supplementary material, figure S1). The predicted Type I error rate was near 0 when population stratification was low and reached its maximum when both *λ*_*S*_ and *λ*_*A*_ were near their maximum values. Although the pattern was qualitatively similar at both sample sizes, the relationship was stronger with 1000 samples. With 1000 samples, the Type I error remained below the *p*-value significance cut-off of 0.0001 over a wider range of the parameter space. However, it rose more quickly with rising *λ*_*S*_ and *λ*_*A*_ and reached a maximum rate that was 10 times higher than with 100 samples. This relationship between population stratification and sample size is consistent with observations in the human population [33].

Using an association strength (*ϕ*) of 0.75 as a default, we found that the simulated SNP promoting human infection was detected 100% of the time if 1000 samples were available (figure 2*d*), but detected only 62% of the time when only 100 samples were available (figure 2*c*). As the effect of the allele promoting human infection was reduced, the power of the method fell as expected (electronic supplementary material, figure S2). Detection of the allele promoting human infection largely depended on its frequency in the population as a whole. The allele was rarely detected when common (9% detection with allele frequencies greater than 75%) but almost always detected when rare (97% detection with allele frequencies less than 25%). Population stratification parameters had a relatively minor, though still significant, effect. Increasing values of *λ*_*S*_ led to a slight decrease in power whereas larger values of *λ*_*A*_ led to a slight increase (figure 2*c*).

### 3.2. Lassa virus data is highly stratified

To better understand how the results of the previous section related to sequence data from real zoonotic viruses, we quantified the extent and nature of stratification within LASV sequences collected from Sierra Leone. Specifically, we studied the phylogenetic and geographical structure of LASV samples using the polymerase gene, which has been associated with virulence [34], species-specificity [35] and host-switching [36] in other negative-sense segmented RNA viruses. There were a total of 92 samples, 67 of which were collected from humans and 25 of which were collected from rodents. We excluded the few cases in which LASV sequences were derived from human infections known to have originated from another human rather than from direct spillover from the rodent reservoir. The data were strongly skewed by geographical source and year of collection. Specifically, 60 of the human samples were collected from a single hospital, Kenema Governmental Hospital (KGH), in the city of Kenema in the Eastern Province between the years 2009 and 2013. Two human samples collected in 1976 were also collected in the Eastern Province. The other five human samples were collected between 1975 and 2000 and have no associated geographical information. Out of the 25 rodent samples, 15 were collected in 2019–2020, mostly from the Northern Province. The 10 remaining rodent samples were collected in 2012 and have no associated geographical information. A map of collection sites can be found in electronic supplementary material, figure S3.

**Figure 3.**
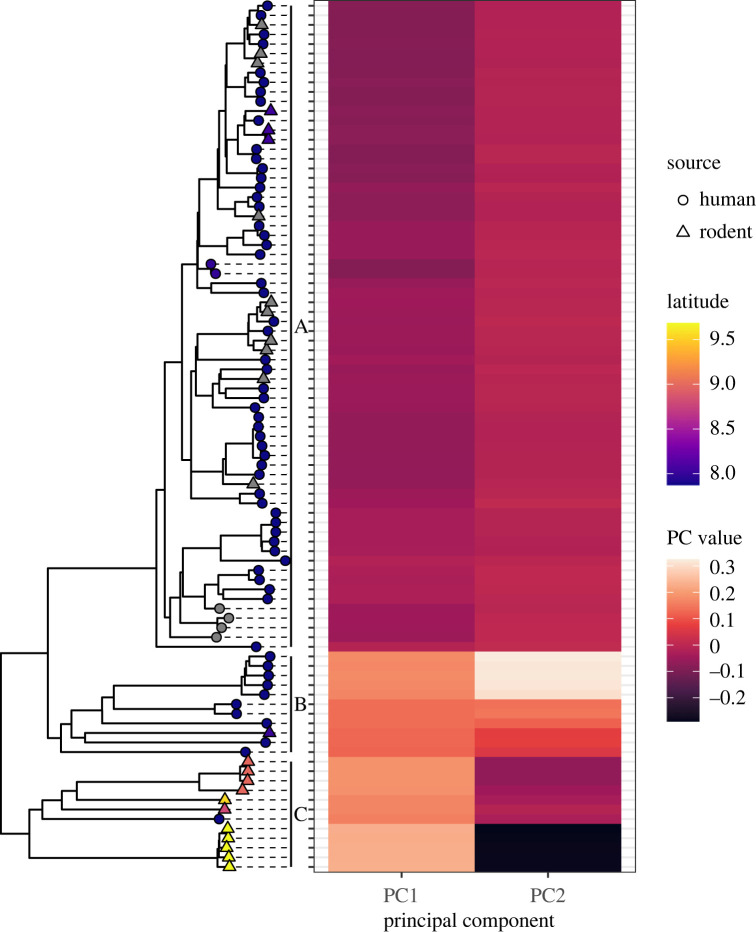
Each tip on the LASV phylogeny was aligned to its PC values calculated by EIGENSTRAT. Three clusters of PC values were formed, each of which corresponded to a clade. Beginning from the top, Clade A had negative PC1 values and intermediate PC2 values; Clade B had positive values for PC1 and PC2; and Clade C had positive values for PC1 and negative values for PC2. Grey tips indicate that no location data were available.

The reconstructed phylogeny could be roughly divided into three major clades (figure 3). The separation between the clades was at least in part due to geographical distance between viral sources. For the 15 rodents with location data, clade membership was associated with sample location of origin. The three samples collected in the Eastern Province were in Clade A, the sample collected in the Southern Province was in Clade B, and all 11 samples collected in the Northern Province were in Clade C. All unlabelled rodent samples were in Clade A.

Understanding the relationship between location and clade was more complicated in humans due to missing data. The human samples collected at KGH were all identified with the hospital’s location, but KGH was the only facility in the country with a Lassa fever treatment ward, and patients were referred there from outlying areas. During the years in which the samples were collected, approximately one-third of Lassa fever patients at KGH had travelled to the Kenema district from another region [37]. Clade A contained most of the KGH samples, as well as other samples, both human and rodent, from the Eastern Province, suggesting those KGH patients were also from the Eastern Province. All but one of the remaining KGH samples were in Clade B. During the years of sample collection, most of the patients who travelled to KGH from outside of the Eastern Province were from Bo, a neighbouring district in the Southern Province [37]. This was the district of origin for the rodent in Clade B. Therefore, while the genetic variation in the KGH samples could have been caused by co-circulation of multiple strains, it is also plausible that each clade corresponded to a province, with Clades A, B and C representing the Eastern, Southern and Northern provinces, respectively.

We next explored the association between the phylogeny and the PCs that EIGENSTRAT calculated during detection of population structure. The PCs produced clusters of similar values which corresponded closely to the phylogeny (figure 3), a finding supported by prior work demonstrating that PC values are a function of time to coalescence [38]. Geographical origin was the only significant predictor of PC values. Despite the 40-year span from oldest sample to newest sample, year of sample collection had no significant effect (*p* = 0.56 for PC1, *p* = 0.84 for PC2 by linear regression) after controlling for latitude.

The strong association between phylogeny and location was consistent with previous observations of spatial genetic variation in LASV [39], with patterns of diversity that vary along east–west and north–south gradients [40–42]. Nearly half of the rodent samples were collected in a different region than the human samples. This was expected to cause systematic differences between the genetic backgrounds of the human and the rodent samples. Further, the phylogenetically clustered distribution of LASV samples from humans and rodents produced a *λ*_*S*_ value exceeding 0.99.

### 3.3. Decreasing population stratification decreased the rate of Type I error in Lassa virus data

Based on our analyses of simulated sequence data and spillover events, we hypothesized that the significant stratification observed in the LASV data from Sierra Leone would lead to elevated Type I error rates. To test this hypothesis, we appended an artificial locus onto each LASV sequence in our dataset and simulated its evolution as described in the methods. Thus, the allelic state at this artificial locus was independent of host identity, as would be the case for a locus with no influence on spillover risk. We did not alter the phylogeny or distribution of hosts within our LASV data, so *λ*_*S*_ was fixed at 0.99.

**Figure 4.**
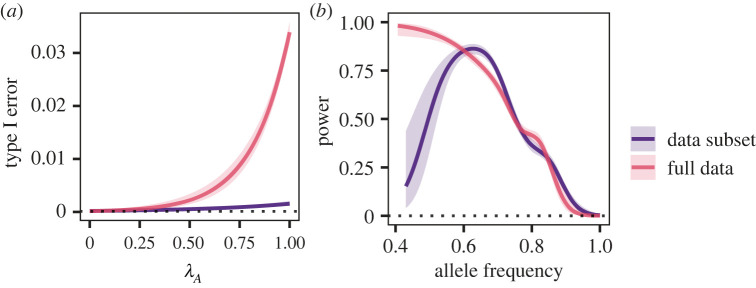
GLM response curves showing (*a*) Type I error rate with and without Clade C. (*b*) The detection rate at *ϕ* = 0.75. The 95% confidence interval is indicated by the shaded area. The dotted line indicates the threshold used to assess GWAS significance, *p* < 0.0001. In (*a*), the GLM was conducted using the mean allele frequency of 0.4 and represents 60 000 replicates for each dataset. In (*b*), the GLM was conducted using the mean *λ*_*A*_ of 0.3 and represents 6000 replicates for each dataset.

As in the simulated data, the Type I error rate at the appended locus increased with its *λ*_*A*_ (figure 4), but even at the lowest *λ*_*A*_, it exceeded the *p*-value threshold by two orders of magnitude. This pattern was qualitatively similar to the simulated data, in which Type I error was elevated across the parameter range of *λ*_*A*_ when *λ*_*S*_ was near 1 (figure 2*a*).

To investigate whether elevated Type I error rates could be managed by reducing stratification within the data, we excluded Clade C from analysis. This decreased *λ*_*S*_ from 0.99 to 0.52. Repeating the simulation analyses on the reduced dataset reduced the predicted Type I error rate (odds ratio = 0.06, 95% CI = 0.05–0.08) such that it did not exceed the *p* cut-off at low *λ*_*A*_ (figure 4). This pattern was qualitatively similar to figure 2*a* for the respective values of *λ*_*S*_.

Because the benefits of reducing stratification by focusing on only a subset of the data may be offset by reduced power, we next evaluated the consequences of Clade C removal for power to detect a simulated locus associated with human spillover. Our results demonstrated that using only a subset of the data did not decrease power at *ϕ* = 0.75 (odds ratio for data subset = 1.07, 95% CI = 0.98–1.18, figure 4*b*). This was qualitatively similar to the simulated data, in which rising values of *λ*_*S*_ were associated with a slight decrease in power (figure 2*c*). As with our earlier analyses of simulated data, power was strongly influenced by the frequency of the allele and decreased as the allele frequency rose. Power dropped with lower values of *ϕ*, especially in the data subset (electronic supplementary material, figure S4).

### 3.4. Genome-wide association studies identified human-associated single-nucleotide polymorphisms in the polymerase gene

**Figure 5.**
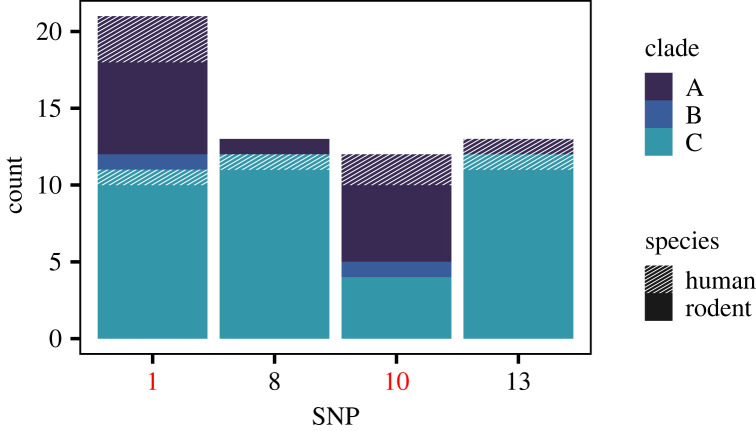
For four representative SNPs, the number of samples in which the minor allele was found is shown, subdivided by clade and species. When Clade C was removed from analysis, only SNPs 1 and 10, shown in red, were detected as significant.

Finally, we performed GWAS on the LASV samples to detect species-associated SNPs. After correcting for population stratification with EIGENSTRAT, no species-associated non-synonymous SNPs were identified in the genes encoding GPC, NP or Z. In the polymerase gene, we identified 13 non-synonymous mutations that were associated with viral host species. However, comparison of the distribution of the SNPs to the phylogeny showed that Clade C was genetically distinct from A and B and was strongly influencing the results (figure 5, electronic supplementary material, figure S5). For example, SNPs 8 and 13 were found in all 12 samples from Clade C but only in a single sample outside of Clade C.

Informed by the distribution of SNPs and the prior set of simulations, we repeated the analysis on a subset of the data with Clade C excluded. Only two non-synonymous SNPs, 1 and 10, were returned as significant (figure 5). SNP 1 is in a linker section that wraps around the polymerase [43]. SNP 10 is located in a putative cap-binding domain [43,44] that is highly analogous to the cap-binding domain in other viruses, such as the bunyaviruses [45,46] and influenza, in which it binds a segment of host mRNA for use as a primer to initiate transcription [47]. In influenza, mutations in the cap-binding domain have been repeatedly linked to host shifts from avian to mammalian infection [36].

## 4. Discussion

Our results show that combining EIGENSTRAT GWAS with biologically informed simulation can provide important insight into the genetic basis of spillover, despite the constraints common to real viral data. Although population stratification and small sample sizes inflated the error rate, this could be partially mitigated by tailoring the analysis to the specifics of the data. Using the LASV data, we found that decreasing population stratification by restricting the analysis to clades with paired rodent and human samples substantially decreased the Type I error rate. In some conditions, this could be achieved without increasing the Type II error rate, despite reducing an already-small sample size. This may be because, given sufficient genetic differentiation, EIGENSTRAT can detect population structure accurately, even with relatively few samples and short genomes [48], but is unable to fully compensate for severe stratification or potential distortion introduced by uneven sampling [38]

Based on these findings, we provide several recommendations for future efforts to identify the genetic basis of spillover. First, population stratification should be minimized at the time of sample collection. For a geographically variable pathogen like LASV, this could be achieved by collecting paired human and animal samples from the same communities and documenting the relevant metadata. However, targeted collection may not be feasible for viral zoonoses, where prevalence within the reservoir is often extremely low. For example, the 15 rodent samples from 2019 to 2020 that were used in this analysis were the product of a collection effort that saw over 1000 rodents trapped across Sierra Leone. Second, when new data collection is impossible, existing data should be filtered to prioritize data quality over data quantity. This informed our decision to limit our analysis to sequences from Sierra Leone. Although there are many human-derived LASV sequences collected in Nigeria, there are only four rodent sequences, none of which were collected from *Mastomys natalensis*, and the nucleotide diversity of LASV is approximately 30% across all lineages [24]. Based on our predictions, including Nigerian sequences in the analysis could decrease the Type II error rate, but probably with the concomitant cost of substantially increasing the Type I error rate. However, any potential benefit would only be realized if the same spillover SNP were present in both lineages. Otherwise, the association would be lost. Third, at the time of analysis, the Type I and II error rates should be assessed using data-informed simulations to identify problematic clades. Finally, because PCA results are vulnerable to population characteristics and decisions made during the analysis [49], systematic evaluation of the likelihood and plausibility of results, which can also be assessed via data-informed simulations, is critical.

Our conceptual approach was the analysis of genetic variants that promote spillover. The SNPs we identified were statistically associated with being sampled from a human host. Though we have interpreted these variants as being those that promote spillover, there are many mechanisms that could produce an association. Only a subset of these mechanisms involve a virus’s ability to switch hosts. For example, a virus like LASV must be shed in sufficient quantities by the rodent reservoir in a form that is contagious to humans and that is robust enough to persist in the environment until a human encounters it [50]. It therefore may be challenging to distinguish mutations that directly potentiate spillover from those that increase the likelihood of human infection, *per se*. Sampling patterns may also introduce bias. A spillover infection must be sufficiently virulent that a patient will seek treatment. In LASV, the clinical manifestations are highly variable, and for every case of haemorrhagic fever, there are many more infections with non-specific symptoms [51]. Because only severely ill patients will present at a hospital where a viral sample can be collected, human samples may represent unusually virulent infections. Even the sample collection protocol could lead to spurious associations, with genetic variation within hosts obscuring patterns of genetic variation between species. Due to organ-specific adaptation, viruses circulating in one organ may carry different SNPs to those from another organ in the same host [52]. If human samples are systematically collected from different organ systems to animal samples, organ-associated SNPs may appear to be species-associated. Other human-associated variants may arise after spillover over the course of adaptation to the novel human environment [53]. For all of these reasons, the identification of candidate SNPs is only the first step towards understanding the genetic basis of spillover.

Here, to guide analysis and sampling of zoonotic viruses in the most general case, we have focused our investigation on the features of the data that are most immediately related to population stratification. There are other factors related to stratification that we did not quantify, such as population differentiation [33] and phylogenetic tree topology [54], either of which could have refined or increased the predictive power of the model. We also made several assumptions about the nature of a spillover mutation that are not universally true. For one, our analysis assumed that spillover was determined by a single mutation with a relatively strong effect. However, epistatic interactions are common in viruses [55], and a spillover trait may be produced by interactions between multiple SNPs, each with little to no effect individually. This analysis did not incorporate linkage disequilibrium and would have been unable to detect weakly associated variants. Although recombination appears rare in LASV, reassortment may occur [24]. Both are common in other viruses [56,57] and have been associated with host shifts [58]. We also assumed the mutation promoting spillover was neutral in our simulated datasets, but adaptations favouring replication in a new host often come at the expense of a decreased ability to infect the original host [59]. Ultimately, this is a correlative method to identify candidate SNPs with the added context of predictions about the robustness of the findings, to guide future investigations.

As the structure of ecosystems is reshaped by climate change and other forms of anthropogenic disturbance, opportunities for spillover are likely to increase [3,5], and the need for proactive approaches to managing zoonotic disease grows increasingly urgent. Because many management techniques, such as wildlife vaccination and culling, are costly and laborious to implement, they must be deployed strategically in areas where the risk of spillover is greatest. Genomic surveillance of spillover variants would be a powerful addition to current approaches that predict risk, such as wildlife surveillance [7,60,61] and risk mapping [62]. By identifying genetic variants that promote spillover, we can maximize the impact of public health interventions by targeting them to regions with the highest genetic risk of spillover.

## Data Availability

Compiled simulation data and LASV data are hosted at OSF (https://osf.io/c7tqp/). The code for simulations, statistical analysis and data visualization is available on Bitbucket (https://bitbucket.org/aobwhitlock/lassaspillover), along with all data required to perform the analyses. Note: due to the possibility of DURC concerns, we have not provided code to create figure 5 and electronic supplementary material, figure S5. The data are provided in electronic supplementary material [63].
